# Phylogeny Drives Large Scale Patterns in Australian Marine Bioactivity and Provides a New Chemical Ecology Rationale for Future Biodiscovery

**DOI:** 10.1371/journal.pone.0073800

**Published:** 2013-09-05

**Authors:** Elizabeth A. Evans-Illidge, Murray Logan, Jason Doyle, Jane Fromont, Christopher N. Battershill, Gavin Ericson, Carsten W. Wolff, Andrew Muirhead, Phillip Kearns, David Abdo, Stuart Kininmonth, Lyndon Llewellyn

**Affiliations:** 1 Australian Institute of Marine Science, Townsville, Queensland, Australia; 2 Western Australian Museum, Welshpool, Western Australia, Australia; Rochester Institute of Technology, United States of America

## Abstract

Twenty-five years of Australian marine bioresources collecting and research by the Australian Institute of Marine Science (AIMS) has explored the breadth of latitudinally and longitudinally diverse marine habitats that comprise Australia’s ocean territory. The resulting AIMS Bioresources Library and associated relational database integrate biodiversity with bioactivity data, and these resources were mined to retrospectively assess biogeographic, taxonomic and phylogenetic patterns in cytotoxic, antimicrobial, and central nervous system (CNS)-protective bioactivity. While the bioassays used were originally chosen to be indicative of pharmaceutically relevant bioactivity, the results have qualified ecological relevance regarding secondary metabolism. In general, metazoan phyla along the deuterostome phylogenetic pathway (eg to Chordata) and their ancestors (eg Porifera and Cnidaria) had higher percentages of bioactive samples in the assays examined. While taxonomy at the phylum level and higher-order phylogeny groupings helped account for observed trends, taxonomy to genus did not resolve the trends any further. In addition, the results did not identify any biogeographic bioactivity hotspots that correlated with biodiversity hotspots. We conclude with a hypothesis that high-level phylogeny, and therefore the metabolic machinery available to an organism, is a major determinant of bioactivity, while habitat diversity and ecological circumstance are possible drivers in the activation of this machinery and bioactive secondary metabolism. This study supports the strategy of targeting phyla from the deuterostome lineage (including ancestral phyla) from biodiverse marine habitats and ecological niches, in future biodiscovery, at least that which is focused on vertebrate (including human) health.

## Introduction

Biodiscovery – or use of biodiversity as a source of innovation for medicine and other useful products - is an ancient concept with nature providing the basis of most early drugs [1]. At the start of the 21^st^ century, an estimated 75% of the world’s population continue to rely on traditional plant-based medicines for primary health care [2], and over 60% of the new chemical entities explored as new drugs in the 25 years to 2007 have their origin in natural products [3]. The vast majority of these innovations are derived from comparatively well understood terrestrial biodiversity, yet of all habitats on the planet, the seafloor holds arguably the greatest potential for biodiscovery because it is the most phylogenetically diverse. It is from the sea that life on earth began billions of years ago, and where 34 of the 36 known phyla of animals remain to this day (with 15 of these exclusive to the world’s oceans [4], [5]. Australia is exceptionally well positioned in the field of marine biodiscovery because it combines a world-class scientific research and development base with immense raw materials within its biodiversity [6]. Australia is one of only 17 recognised megabiodiverse countries primarily based on its highly biodiverse and endemic terrestrial flora and fauna [7], [8], but this trend is also mirrored in the sea.

With an immense 14 million square kilometre ocean territory spanning 36000 km of mainland coastline plus some 12000 islands, Australia’s marine territory straddles three major ocean systems, multiple palaeontological origins, and a plethora of diverse and unique biophysical features along a latitudinal range from the tropics to Antarctica [9]. Between these extremes lies a diversity of habitat types further elaborated by transition and overlap zones, where otherwise distinct species assemblages mix to create a high incidence of endemic species, as has been described on the Western Australian coastline [10]. While the full extent of Australian marine biodiversity remains relatively unexplored [9], several marine biodiversity hotspots including centres of endemicity have been recognised, especially in coral reefs [11], [12], the temperate coastline [13] and the Great Australian Bight off the coast of South Australia is now known to support one of the world’s most diverse soft sediment ecosystems [14]. There have been reports on the high species diversity of sponges in the north west [15], [16], [17], [18], and in the deep sea off the south west [19], [20], and the Great Barrier Reef [18].

Research on natural products in Australian marine organisms has a 50+ year history, with the earliest publications in the 1960s addressing toxins in cyanobacteria [21] and useful photosynthetic pigments from marine plants [22]. The immense natural products potential of Australian marine biodiversity has been highlighted in reviews. Volkman [23] outlined a wide range of natural products from algae, microbes, ascidians, bryozoans, corals and sponges and their application to diverse commercial sectors including pharmaceuticals, sunscreens, functional foods, antifouling, and coral sperm attractants. Ghisalberti and Jefferies [24] reported over 110 compounds identified from various organisms including sponges, algae, seagrasses, echinoderms, cyanophytes and sediments, from Western Australia alone. Australia received its first major impetus for pharmaceutically oriented research in 1974 with the establishment of the Roche Institute of Marine Pharmacology in New South Wales [23], [25]. Since then, significant collection and biodiscovery research effort has been supported by the USA government through the National Cancer Institute; industry including pharmaceutical and agrichemical interests; and the Australian government through universities and public research institutions [6], [23], [26].

While the global marine biodiscovery research effort had identified 18000 new chemical entities by 2010 [4] with 10000 of these since 1990 from invertebrates alone [5], and despite the promising potential for Australia to feature in the global marine biodiscovery effort, contributions from Australian marine biodiversity are conspicuously underrepresented in the literature in a trend that appears to be worsening. During the decade 1980–1990, almost 7% of the over 4000 articles linked through the marine natural products literature database *Marinlit* were based on Australian marine biota, however this figure dropped to 2% of the over 10000 articles published during the decade to 2010 [27]. This trend varies between Australian jurisdictions, but is worst in Western Australia which contributes over one third of Australia’s marine estate, yet only 2% of the Australian marine natural products literature to 2010 related to marine biota from that State [27].

One often cited reason for this downturn is a legal issue. Australia is a party to the Convention on Biological Diversity which came into force in 1993 [28] and created a legal requirement to facilitate access to biodiversity for biodiscovery research whilst ensuring equitable sharing of the benefits arising from this use [29]. Governments of all nine Australian jurisdictions agreed to develop a Nationally Consistent Approach (NCA) to development of domestic laws to consistently implement the access and benefit sharing requirements of the CBD across Australia, however while three jurisdictions (Queensland, Northern Territory, and the Commonwealth governments) have implemented new or amended customized legislation, most jurisdictions have not [29], [30]. Difficulty in domestic implementation of the CBD is widespread outside Australia [31], and is at least in part blamed for a global downturn in commercial uptake of natural products research. Butler [32] reported no new natural-product templates or novel structures had been discovered since 1996 that resulted in compounds entering clinical trials.

Thus, jurisdictions without clear biodiscovery laws are at a significant disadvantage in biodiscovery research and its commercial potential. For example, two particularly promising Western Australian vacuolar ATPase inhibiting anti-cancer drug candidates emerged from a major collaboration between AIMS and the National Cancer Institute, but were not progressed further due to issues associated with access permits. These are the salicilihalamides from the sponge *Haliclona* sp [33], [34] and the lobatamides from the ascidian *Aplidium lobatum* [35]. Western Australia is also the origin of the soft coral *Elutherobia* sp, from which the diterpene glycoside eleutherobin was first isolated and identified as a potent tubulin polymerization agent with significant anti-cancer potential [36], [37]. Due to difficulties in obtaining permits for recollection, this compound was subject to an intensive synthetic research effort based in the USA [38], [39], [40]. Simpler analogues were synthesised [41], [42] and explored for therapeutic potential [43]. Eleutherobin was also subsequently found in a more readily accessible natural source, the Caribbean coral *Erythropodium caribeaorum* [43], which is amenable to aquaculture [44]. Thus, while options have been identified for a supply pathway to support development of this particular therapeutic lead, it has come at a considerable additional overhead (itself a disincentive to activity in this field) and none of the options involve biota, research or industry involvement in the original source jurisdiction of Western Australia.

The Nagoya Protocol is a new legally binding protocol within the CBD which was opened for signature in 2011 [45]. When the Nagoya Protocol comes into force (possibly in 2013) it has the potential to resolve historical legal uncertainties, if domestic regimes support its implementation [45]. Simultaneously, a renaissance of interest in natural products has been reported, particularly as a scaffold for new therapeutics [3], [46].

Previous workers have sought to relate specific bioactivity to ecological function and small scale environmental gradients [47], [48] and use ecology and biology to explain intraspecific variability [33], [47], [48], [49], [50], [51], [52], [53]. However, there are no published studies that comprehensively integrate a diverse range of biodiversity and bioactivity measurements across vast bioregional and phylogenetic gradients. This paper attempts such a broad-scale overview of bioactivity across Australia, to support the re-emergence of the global biodiscovery opportunity with a reassessment of the potential for marine biodiscovery in Australia.

We analysed data on cytotoxic, antimicrobial, and central nervous system (CNS) protective bioactivity within a major Australian marine collection facility at the Australian Institute of Marine Science (AIMS), the AIMS Bioresources Library. Trends in bioactivity are explored with respect to the taxonomy and phylogeny of the samples and the bioregions where they were collected. An appraisal of Australian marine biodiversity as a source of pharmaceutically interesting bioactivity in three therapeutic areas of interest for human health is provided. In addition, this study includes an interpretation of these bioactive trends which qualifies the bioassays used with their ecological relevance, to provide an insight into the chemical ecology of Australia’s mega-biodiverse marine estate, and the possible drivers of secondary metabolite production.

## Materials and Methods

**Figure 1:**
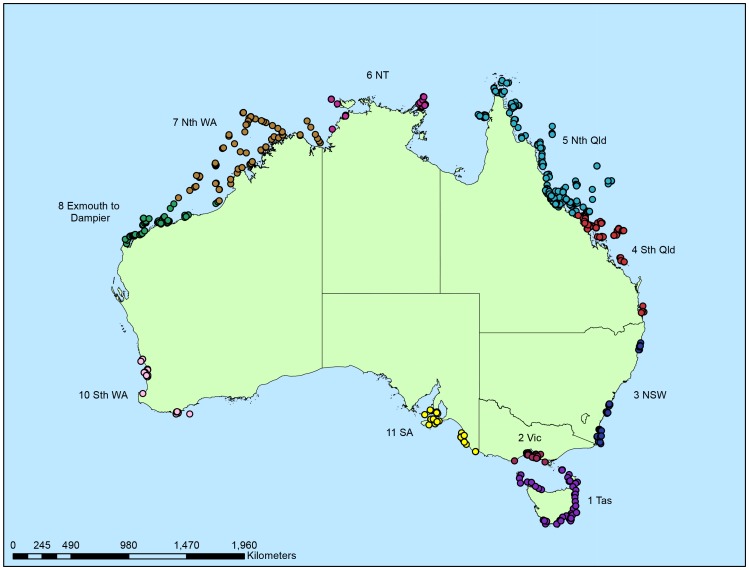
Collection locations (1286 sites) of samples used in this study. The sites are colour coded by the bioregions used in the spatial analysis. Note that there is no bioregion number 9 (excluded due to limited samples).

Marine samples included in this study were collected from around the Australian coastline (Figure 1) via hand collection by divers or the use of trawl equipment. Appropriate collection permits from relevant government agencies were obtained, and the position of each collection site was recorded by radar-plot (pre-1994) or GPS. Full details of collection permits are included in file S1. Biodiversity data collected for each uniquely identified sample included taxonomic assignment (to species if available); and collection data including date, depth, and specific location. Each collection location was assigned to a bioregional area. Figure 1 shows the 1286 collection locations of the samples in this data set, colour coded according to the bioregions used in the analyses undertaken in this project.

The database associated with the Bioresources Library at the Australian Institute of Marine Science (AIMS) is relational and integrates the collection and biodiversity data associated with uniquely identified samples, with data from extract screening and biodiscovery history. This database was interrogated to compile a subset of data for uniquely identified samples collected from ten Australian bioregions identified in Figure 1, which had been screened in the bioassays described below.

Data from several bioassays were included in this study and were grouped into the following three functional categories for the purpose of further analysis of bioactivity: cytotoxicity (against 3 human tumor cell lines); antimicrobial activity (against 4 surrogates of microbial pathogens of humans); and CNS protection (inhibition of nNOS and the calcium channel). Experimental detail of sample processing and each of the bioassays is included in file S2. Tables A–C in file S2 list the number of samples screened in bioassays of each of these three bioactivity categories, within each phylum and bioregion. Collectively, the analyses in this paper combine over 18000 individual screening results across 10 Australian marine bioregions and 13 phyla.

### Statistical Analysis of Spatial and Taxonomic Patterns in Bioactivity

Each dataset was compiled and analysed separately for each of the bioassay categories.

The measure of bioactivity for each sample in each bioassay was normalized to the relevant control (expressed as a percentage of the value of the control), such that a lower result indicated a more bioactive sample. Within each bioassay category, the result for each bioassay was treated as a repeated measure of bioactivity in that category.

The overall mean and standard deviation were calculated, and the threshold for attribution of ‘bioactivity’ was set as equal to or lower than one standard deviation from the mean. While this is a more conservative threshold than that used by other workers who have focused on identification of pharmaceutical leads (eg the five standard deviation threshold used in [54]), it was chosen in this study as a more appropriate measure of ecologically relevant bioactivity, as lower concentrations are more likely to be encountered *in situ*. Each sample was assigned as ‘active’ or ‘non-active’ in each bioassay category, with respect to this threshold. The number of active samples in each phylum/bioregion combination was then expressed as a percentage of the total samples tested in each data cell (bioregion × phylum). The following method was then used to investigate patterns in bioactivity according to phylum and bioregion, and their interactions.

Relative bioactivity of phyla across the bioregion codes were predicted using hierarchical mixed effects models using actual bioassay results, and subsequent singular (simple main effects) regression models using the percentage bioactivity results for each data cell (bioregion × phylum). These analyses were fitted in a Bayesian framework using non-informative normal prior probability distributions for each of the coefficients, and broad uniform distributions for standard deviations [55]. This method is robust for any potential bias from imbalanced sample sizes between the phyla. Each simple main effects model comprised of a fixed factor of either bioregion or phylum. Inference was based on 95% Bayesian credibility intervals (CI) of cell means predicted from posterior distributions of model parameters derived via Markov-chain Monte Carlo (MCMC) sampling using Open BUG’s [56] interfaced with BRugs package of R version 2.13.1 [57]. Model convergence was assessed visually [58] for three simultaneously running Markov chains of 20 000 iterations, following a 5000 iteration burn-in. Sample independence for each parameter was confirmed via autocorrelation values less than 0.1.

To investigate the scope of finer phylogenetic scaling on the spatial patterns of bioactivity, the Bayesian Hierarchical models were refitted for antimicrobial bioactivity aggregated at the lower-order taxonomic level of genus.

**Figure 2:**
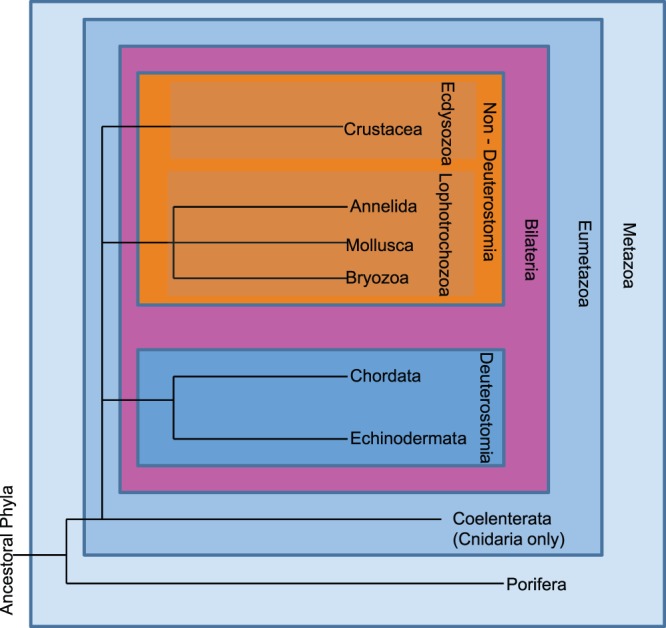
Phylogenetic position of the animal Phyla examined in this study. This scheme follows a modern synthesis based largely on molecular data, reported by Halanych [59] and further resolved by Philippe et al [66]and Srivastava et al [67]. This figure is restricted to the position of phyla considered in the present study only. This figures establishes the colour scheme used for animal phyla throughout figures 3–8 (Light blue, mid blue, dark blue and orange represents Early-Metazoan, Early-Eumetazoan, Deuterostome and non-Deuterostome ineages respectively.).

To assess bioactivity trends with metazoan phylogeny, the animal phyla examined were categorized as from the deuterostome lineage (including, for this analysis, the ancestral phyla Porifera and Cnidaria) or non-deuterostome lineage, following Halanych [59], whose modern animal phylogenetic synthesis is based on molecular data and is summarized in figure 2.

## Results

**Figure 3:**
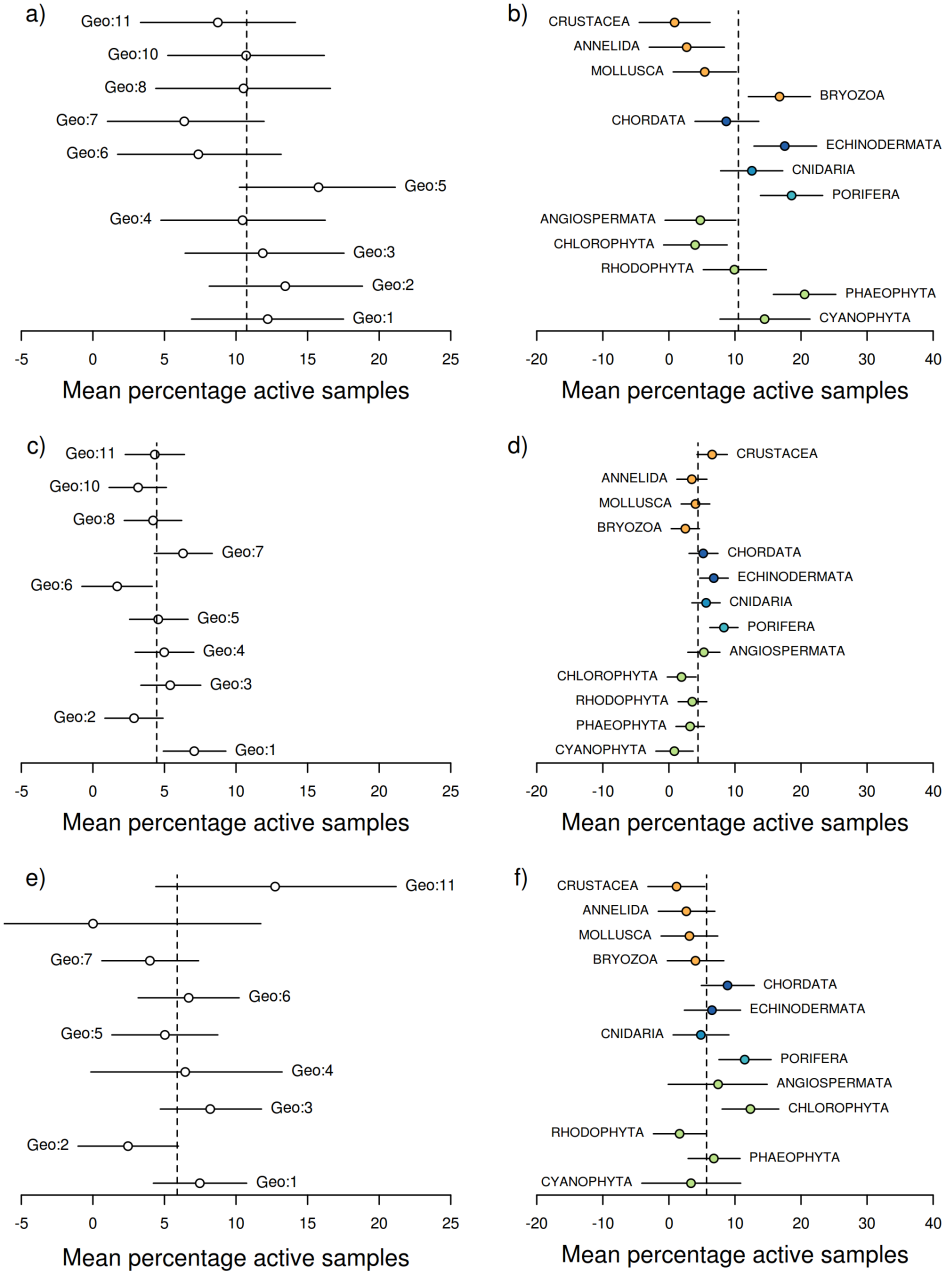
Results of the Bayesian Hierarchical analyses. Mean (±95% CI) predicted marginal means of percentage of bioassay samples assigned as active (activity score lower than one standard deviation below the mean) according to bioregion and Phyla (respectively), for the three bioassay types: a) and b) Cytotoxicity; c) and d) antimicrobial; and e) and f) CNS Protective. Green, light blue, mid blue, dark blue and orange symbols represent plant/algae; Early-Metazoan, Early-Eumetazoan, Deuterostome and non-Deuterostome lineages respectively. This colour scheme is used throughout figures 3–8.

**Figure 4:**
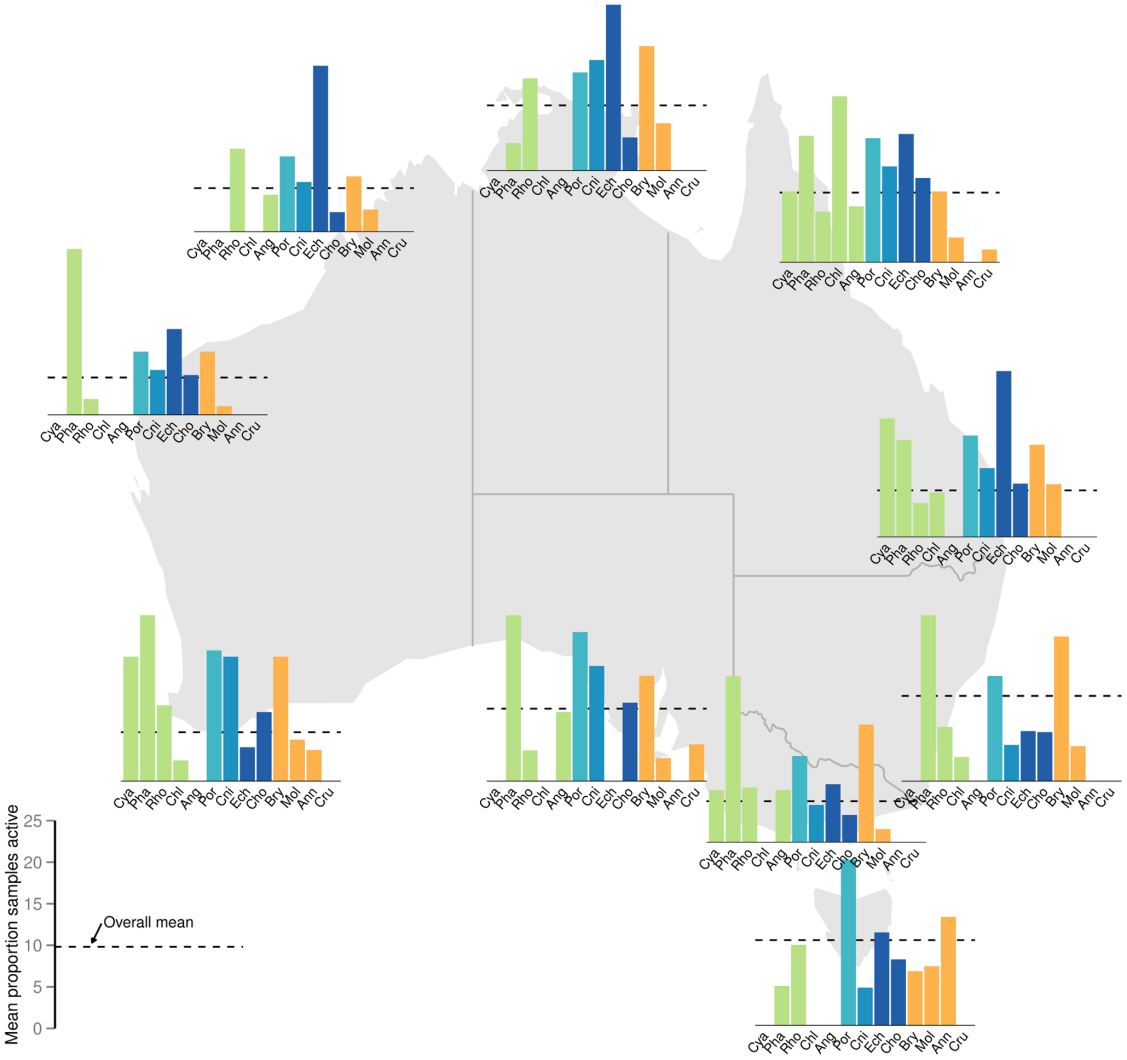
Prevalence of cytotoxic active samples by phylum, mapped over bioregions. Percentage of samples in each Phylum with cytotoxicity bioactivity greater than the threshold (one standard deviation from the mean) mapped over bioregions considered in this study. Phylum abbreviations are: Cya = Cyanophyta; Pha = Phaeophyta; Rho = Rhodophyta; Chl = Chlorophyta; Ang = Angiospermata; Por = Porifera; Cni = Cnidaria; Ech = Echinodermata; Cho = Chordata; Bry = Bryozoa; Mol = Mollusca; Ann = Annelida; Cru = Crustacea. Green, light blue, mid blue, dark blue and orange symbols represent plant/algae; Early-Metazoan, Early-Eumetazoan, Deuterostome and non-Deuterostome ineages respectively. This colour scheme is used throughout figures 3–8.

**Figure 5:**
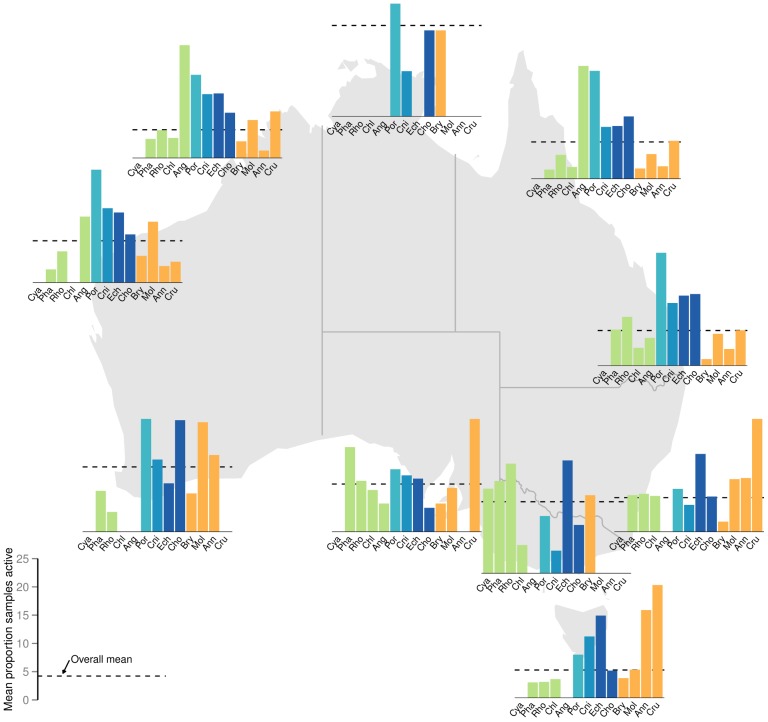
Prevalence of antimicrobial active samples by phylum, mapped over bioregions. Percentage of samples in each Phylum with antimicrobial bioactivity greater than the threshold (one standard deviation from the mean) mapped over bioregions considered in this study. Phylum abbreviations are: Cya = Cyanophyta; Pha = Phaeophyta; Rho = Rhodophyta; Chl = Chlorophyta; Ang = Angiospermata; Por = Porifera; Cni = Cnidaria; Ech = Echinodermata; Cho = Chordata; Bry = Bryozoa; Mol = Mollusca; Ann = Annelida; Cru = Crustacea. Green, light blue, mid blue, dark blue and orange symbols represent plant/algae; Early-Metazoan, Early-Eumetazoan, Deuterostome and non-Deuterostome ineages respectively. This colour scheme is used throughout figures 3–8.

**Figure 6:**
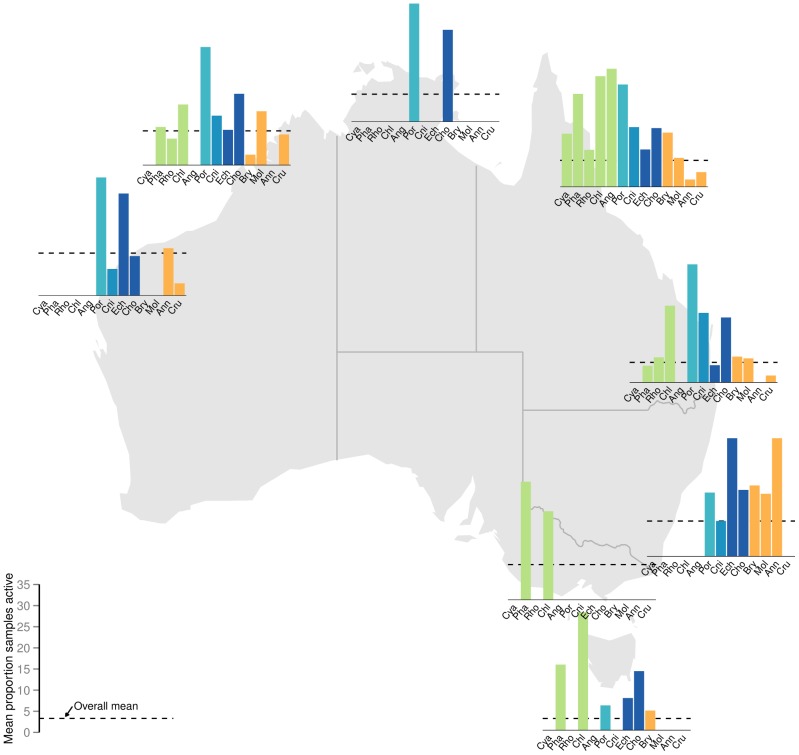
Prevalence of CNS-protective active samples by phylum, mapped over bioregions. Percentage of samples in each Phylum with CNS-protective bioactivity greater than the threshold (one standard deviation from the mean) mapped over bioregions considered in this study. Phylum abbreviations are: Cya = Cyanophyta; Pha = Phaeophyta; Rho = Rhodophyta; Chl = Chlorophyta; Ang = Angiospermata; Por = Porifera; Cni = Cnidaria; Ech = Echinodermata; Cho = Chordata; Bry = Bryozoa; Mol = Mollusca; Ann = Annelida; Cru = Crustacea. Green, light blue, mid blue, dark blue and orange symbols represent plant/algae; Early-Metazoan, Early-Eumetazoan, Deuterostome and non-Deuterostome ineages respectively. This colour scheme is used throughout figures 3–8.

**Figure 7:**
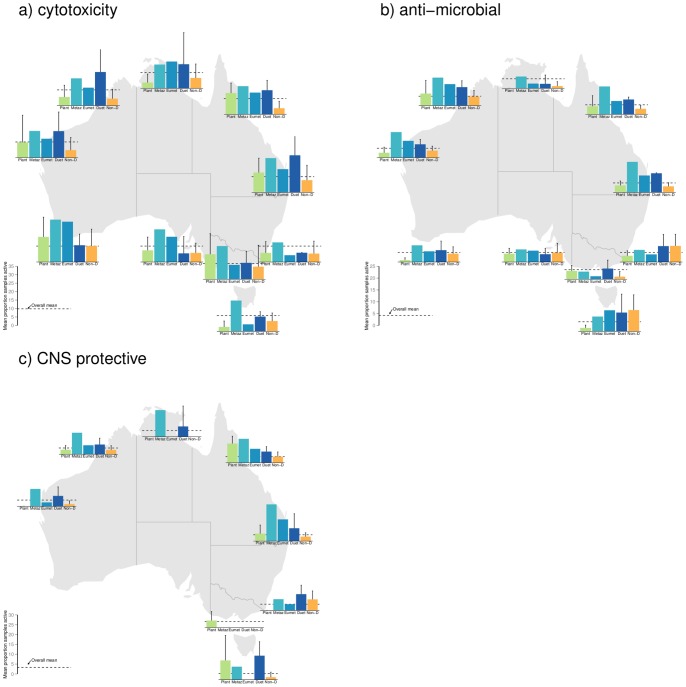
Prevalence of bioactivity by phylogenetic lineage, mapped over bioregions. Mean percentage (±95%) of a) cytotoxicity; b) antimicrobial and c) CNS protective bioactive samples (less than one standard deviation below the mean) amongst phyla of each phylogenetic lineage, mapped over bioregions considered in this study. Green, light blue, mid blue, dark blue and orange symbols represent plant/algae; Early-Metazoan, Early-Eumetazoan, Deuterostome and non-Deuterostome ineages respectively. This colour scheme is used throughout figures 3–8.

The results of the Bayesian analyses are presented in the plots of predicted marginal means of percentage of bioactive samples across bioregion and phylum for each bioassay category (Figure 3). As bioactivity (proportion of samples displaying activity levels above specific threshold levels) associated with each bioregion and phylum combination is necessarily based on an accumulation of samples, formal statistical analyses of bioregion by phyla interactions was precluded. Nevertheless, Figures 4, 5 and 6 depict the distribution of percentage active samples in each phylum, mapped over bioregions (for cytotoxicity, antimicrobial and CNS-protective bioassay categories respectively) and thereby provide graphical representations of such interactions. Further, these results are compiled by phylogenetic lineage and mapped over bioregions for each bioassay category (Figure 7).

Overall bioactivity amongst the bioregions differed only slightly between the three bioassay types, with no bioregion showing a higher than average percentage of bioactives in any bioassay category (Figure 3 a, c, e). The marginal mean percentage of active samples (CI 95%) for phyla in each bioassay category is presented in Figure 3 b, d and f. Porifera (sponges) was the only phylum to consistently show a significantly higher percentage of bioactive samples compared to the mean in all three bioassay categories. Sponges were also the most consistently bioactive phylum across all bioregions (Figures 4, 5 and 6). Most other phyla showed a similar or lower percentage of bioactive samples compared to the mean (CI 95%), with the following exceptions (Figure 3 b, d, f).

In the cytotoxicity bioassays, the deuterostome phylum Echinodermata and early-Eumetazoan phylum Cnidaria, showed a higher percentage of cytotoxic samples (Figure 3b) primarily due to high cnidarian cytotoxicity in south Western Australia, South Australia, and northern eastern Australia; and high echinoderm cytotoxicity in northern and eastern Australia (Figure 4). The non-deuterostome Bryozoa, and the brown algae (Phaeophyta), also showed a higher percentage of cytotoxic samples (Figure 3b). In the case of brown algae, this was primarily due to higher bioactivity in temperate and southern Australia. In Bryozoa, this was due to a higher than average percentage of cytotoxic actives in southern and eastern Australian bioregions (Figure 4). Green algae (Chlorophyta) were significantly more bioactive in the CNS-protective actives (Figure 3 f), primarily due to a higher than average percentage of actives along the Queensland coast (Figure 6).

**Figure 8:**
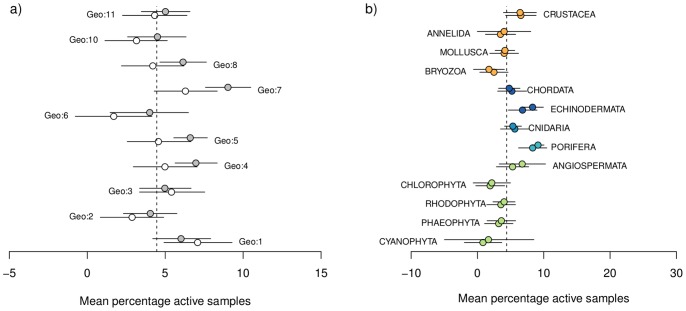
Results of the Bayesian Hierarchical analyses of data attributed to phylum vs genus. Mean (+– 95% CI) predicted marginal means of percentage of bioassay samples assigned as active (activity score lower than one standard deviation below the mean) in antimicrobial bioassays, according to bioregion (left hand figure) and Phyla (right hand figure). Results for analyses based on Phyla classification is plotted offset to the analyses based on Genus classification. Green, light blue, mid blue, dark blue and orange symbols represent plant/algae; Early-Metazoan, Early-Eumetazoan, Deuterostome and non-Deuterostome ineages respectively. This colour scheme is used throughout figures 3–8.

The antimicrobial bioactivity analyses represented in Figure 3 c and d were repeated using samples aggregated at the lower-order taxonomic classification of genus. Figure 8 contrasts the marginal mean percentage bioactivities of phylum based and genus based analyses. The proportion of antimicrobial bioactivity within each taxonomic division derived from genus level analysis was very consistent with those from the phylum level analysis, and the spatial patterns of bioactivity remained broadly similar (albeit slightly lower).

## Discussion

This study found that phylogentic relatedness, or phylum classification, is the major determinant of level of bioactivity (Figures 3–6). We propose that this is based on evolution of similar metabolic pathways and capacity for the production of secondary metabolites within a phylum, as members of a phylum share a common morphological, developmental and evolutionary relatedness [60].

Porifera were the most bioactive phylum, with the highest percentage of samples showing activity across all three bioassay types and most bioregions (Figure 3–6). This finding is consistent with those of previous workers, who have found that sponges are a rich source of novel secondary metabolites and bioactivity across a wide range of bioassay targets [23], [61], [62]. Since the field of marine biodiscovery and natural products chemistry began, sponges have been highlighted by many authors as the most bioactive marine macro-organism phylum [23], [63], [64], and they accounted for half of the marine natural products that had progressed to pre-clinical or clinical trials as new anti-cancer agents in 2005 [65].

To further explore the concept of phylum level attributes explaining the potential for secondary metabolism and consequent bioactivity, this study assigned each animal phylum into one of four main phylogenetic lineages according to a modern phylogenetic synthesis based largely on molecular data [59], [66], [67]. The deuterostomes and the ancestral early-metazoans (Porifera) and early eumetazoans (Cnidaria) typically had a higher percentage of bioactive samples, compared to non-deuterostome samples, in most bioregions and bioassay categories examined (Figure 7).

The higher percentages of bioactive samples found in the deuterostomes and their ancestors may reflect a bias in this study, due to the use of bioassays designed for therapeutic targets of relevance to animals on the deuterostome lineage (mammals (humans)). Our results cautiously support the idea that the core metabolic attributes of the ancestral phyla Porifera and Cnidaria, have been lost from phyla in the non-deuterostome lineage. It would be useful to further explore this suggestion through a review of metabolic studies in the non-deuterostome lineage. It would also be informative to expand a phylogenetically based bioactivity and secondary metabolism review to include measures of activity in bioassays with greater ecological relevance, to augment the human-pharmaceutical bias in this present study. If the results show similar patterns this would suggest secondary metabolism as a strategy for marine benthic success.

The phyla attributed in this study to the deuterostome lineage and the ancestral phyla (Porifera and Cnidaria) are well reported in the biodiscovery and bioactivity literature [23], and have delivered a significant proportion of pre-clinical or clinical marine cytotoxic anti-cancer candidates [65]. With the exception of Echinodermata, these phyla are traditional sources for marine natural products research [63], perhaps for their typically high biomass and therefore collectability and convenience as well as for their bioactivity. The four phyla in this lineage were cited as source organisms in over 80% of the marine natural products literature from 2001–2005 (expressed as a percentage of citations from 1965 to 2005) [63].

Plants and Bryozoa also featured in the cytotoxicity bioassays, returning a high percentage of samples more active than the threshold. The Bryozoa also have a long history of documented secondary metabolism (14% of citations reported in [63]), and until recently they were historically associated with the deuterostome lineage [59]. Plants are also well represented in the marine natural products literature (over 60% of citations reported in [63], [68]), and within Australia macro-algal biodiversity is particularly high especially along the southern coastlines [69], [70].

Cytotoxicity bioassays used in this study measured the ability of crude extracts to inhibit the growth of clinically relevant human (mammalian) cancer. Toxicity is well reported in the literature to provide important ecological functions especially for sessile invertebrates and plants. Putative and demonstrated roles of broadly toxic metabolites include anti-fouling, chemical defense against predators, protection of reproductive products, and competition for space [23], [47], [49], [50], [53], [62], [71].

While the targets of the bioassays used in this study were inspired by relevance to human therapeutics, the results can be cautiously discussed in an ecological context, with consideration of the different mechanisms of action. In general, the incidence of cytotoxicity was higher and more widespread across all phyla examined (Figure 4), compared to the other two bioassay categories of antimicrobial and CNS protective bioactivity (Figure 5–6). This trend may be seen as a toxicity gradient from gross toxicity, to more selective and targeted secondary metabolism which was measured in the latter two categories. These activities were more restricted to the deuterostome phyla, the ancestral phyla (Porifera and Cnidaria), and some plants (Figure 5–6).

The antimicrobial bioactivity reported here was also widespread among bioregions and again was dominated by the deuterostomes plus the ancestral phyla, although was less prevalent than cytotoxicity (Figure 7). These antimicrobial bioassays measured the ability of extracts to inhibit growth of 4 microbial cell lines which were surrogates of human pathogens. However, members of each of the microbial genera have been recorded as occurring in the sea [72], [73], [74]. There is a very strong ecological rationale for sessile organisms to invest in antimicrobial secondary metabolites to protect themselves against the large diversity and immense biomass of potentially pathogenic microorganisms present in seawater [75], [76]. Antimicrobial secondary metabolites have been recorded from a wide range of ancestral and deuterostome phyla, including Porifera [77], Cnidaria [78], [79], [80], and Chordata [81]. Many marine organisms, including ascidians (Chordata) [82], sponges (Porifera) [77], [83] and soft corals (Cnidaria), have been shown to harbour species-specific assemblages of microorganisms and in some cases these have been shown to play a role in protecting the ‘host’ organism against other microbes [77].

CNS protective bioactivity was much less widespread among bioregions but always a feature of deuterostomes and the ancestral phyla, especially Porifera (Figure 7). The CNS bioassays measured very specific bioactivity as outlined below. Thus, while the CNS actives were fewer in number, there is a much greater likelihood that the molecules responsible for the bioactivity were true secondary metabolites with a targeted, specific function.

The CNS bioassays used in this study measured inhibition of neuronal nitric oxide synthase (nNOS) and the calcium channel. nNOS is a catalyst for the biosynthesis of nitric oxide (NO), which is an important messenger chemical with numerous functions including neurotransmission. However, it is a reactive molecule with one unoccupied electron, and its excessive production causes many diseases including post-stroke damage and some mental illness. As the neuronal form of NOS is a calcium regulated enzyme, selective inhibition of both the calcium channel and nNOS has become an important therapeutic target [84], [85], [86].

While this study measured bioactivity relevant to diseases of the human central nervous system, an ecological discussion of these results is relevant even for sponges with their elementary integrative signaling system devoid of synaptic connections [87]. This is because the very specific cell-signaling activity measured here involves receptor sites and metabolic processes that have been demonstrated to occur in sponges and other invertebrates [88], and have even been postulated as early evidence of nervous system evolution [89], [90]. Calcium dependant nNOS has been implicated in maintenance of Cnidarian-dinoflagellate symbioses [91] and control of the temperature signaling cascade in sponges [89], and nNOS inhibition has been demonstrated by select aplysinopsins –marine indole alkaloids found in certain sponges and corals [92], and the eusynstyelamides found in an ascidian [93].

The overview of patterns in bioactivity presented here has attempted to qualify the ecological relevance of the bioassays utilized. However, it is also important to consider the concentration at which extracts exhibit the activity and the duration of the experiments, with respect to the concentrations and duration of exposure likely to be found *in situ* [71], [75], [94]. While the present study used a relatively conservative threshold for bioactivity in an attempt to compensate at least partly for the presumed lower concentrations *in situ*, such a scalable comparison was not possible because individual extract concentration data was not available.

Notwithstanding some phylum-specific interactions shown in figures 4–6, none of the bioregions examined in this study showed a significantly higher percentage of bioactive samples. Thus – this study does not directly support the notion of any bioactivity ‘hot spots’, despite the high level of endemism and biodiversity hotspots reported from Australia’s marine environment [9], [11], [12], [13], [14], [15], [16], [17], [20], [95]. Further, the primary importance of phylum level characteristics in determining potential for secondary metabolism is supported by the patterns of bioactivity remaining fairly consistent spatially despite finer taxonomic resolution to genus (Figure 8). We propose a hypothesis that secondary metabolism and resulting bioactivity is primarily driven by high level taxonomic classification as a proxy for the metabolic potential bestowed by phylogeny which members of a phylum have in common, and secondarily due to ecological circumstance and the presence or absence of ecological triggers for metabolism to occur.

Individual ecological circumstance was not incorporated in the current analyses, however significant variability in metabolism and resulting bioactivity has been demonstrated by many workers to be heavily influenced by immediate ecology and environmental conditions, even within a species. There are reports of intra-specific variability in bioactivity and metabolite production across spatial and temporal scales [33], [96], environmental factors such as growth form, spatial competition, seasons, and depths [48], [50], [51]; and biotic pressures including life history stage, size, and light availability [47], [49], [52] and physical damage [53]. Production of secondary metabolites in response to environmental stimuli can be very rapid (and hence highly variable through time), with activated chemical defense occurring within a few seconds in one sponge [53], [62]. Secondary metabolism is energy intensive, and a negative correlation between bioactivity and sponge growth has been demonstrated in one sponge, supporting a trade-off in investment in energy for both mechanisms [48]. Thus, organisms will probably not commit energy to producing a compound unless the stimuli for its purpose is present, and the above evidence for environmental control over secondary metabolism and related bioactivity is not surprising.

Given the correlation between phylogeny and bioactivity shown in this study, we suggest that future biodiscovery research would benefit from a taxonomic rationale, by selecting samples from phyla with metabolic attributes that parallel those of the target system. Further, the importance of deuterostome phyla combined with their ancestral phyla, with respect to biodiscovery projects with human disease relevance, highlights the importance of not just targeting, but also protecting these groups in marine conservation planning. To truly preserve this potential for future generations, these phyla must be maintained in their fullest ecosystem integrity, to promote maximum opportunity for secondary metabolism expression, as discussed above.

In conclusion, the results of this study propose that bioactivity, and by extension biodiscovery opportunity, is driven primarily by diversity in high-order taxonomy (and therefore phylogeny) and secondarily by the ecological opportunity to elaborate metabolic potential. Australia is fortunate to have both a highly diverse marine biota and a high diversity of habitat types. High taxonomic diversity is seen in the deuterstome phyla and their ancestors such as the Porifera [15], [16], [17], [19]. This diversity is overlaid with diverse and unique biophysical features through latitudes that span from the tropical Indo-West Pacific to the cool temperate Southern Ocean, including unique combinations of overlap and zones of transition with mixed fauna and high endemism [10]. Thus, Australia is endowed with both diverse metabolic machinery (taxonomic diversity), and an unexplored plethora of environmental and ecological stresses and stimuli to drive secondary metabolite production.

A global downturn in natural products research coincided with the introduction of combinatorial chemistry as a source of metabolite diversity for discovery programs, but this era has come to a disappointing end, with only one lead emerging as a drug candidate [3]. Drug discovery pipelines are again turning to natural products as a source of greater molecular diversity [32], [97]. However, it is the uniqueness of many natural product core structures that makes them of interest as templates or scaffolds [98] about which synthetic studies may generate efficacious and more accessible (to synthesis) compounds [32]. This is the approach that has been taken in the case of eribulin, a synthetic analogue of the sponge derived halichondrin B which is now approved for clinical use against metastatic breast cancer [46], [99]. With issues of legal certainty close to resolution, the field of marine biodiscovery in Australia is well poised to realize its potential at the center of a global renaissance in natural products discovery.

## Supporting Information

File S1 **Detail of field collection permits.**
(DOCX)

File S2 **Experimental detail for sample processing and biossays; and number of samples in each bioassay × bioregion × phylum combination.**
(DOCX)
