# Supplementary material for: Phylogeny Drives Large Scale Patterns in Australian Marine Bioactivity and Provides a New Chemical Ecology Rationale for Future Biodiscovery
Source: PLoS One. 2013 Sep 5;8(9):e73800. doi: 10.1371/journal.pone.0073800 (PMC3763996; doi:10.1371/journal.pone.0073800)
Supplement: File S1 — Detail of field collection permits. (DOCX) [file pone.0073800.s001.docx]

**Phylogeny drives large scale patterns in Australian marine bioactivity and provides a new chemical ecology rationale for future biodiscovery.**

**File S1:** Supporting Information – details of permits and agreements that provide permission for the field studies including collection of samples used in this manuscript.

| Permitting Agency | Relevant legislation | Reference No |
| --- | --- | --- |
| GBRMPA | GBRMPA Regulation | G87/109 |
| GBRMPA | GBRMPA Regulation | G87/293 |
| GBRMPA | GBRMP Regulations; Marine Park Regulation 1983 (Qld) | G88/171a & G88/171b |
| Qld Department of Primary Industries | Qld Fisheries Act 1976-1984 (section 58) | 1780 |
| NSW Department of Agriculture | Fisheries and Oyster Farms Act 1935 | F87/1859 PJE;HB |
| Dept of Conservation Forests and Lands | Fisheries Act (1968) | 89-R-38 |
| Dept of Sea Fisheries Tasmania | Fisheries Act 1959 Section 52 | 7/1/14/63 |
| Dept of Sea Fisheries Tasmania | Fisheries Act 1959 Section 52 | 7/1/14/63 |
| SA Dept of Fisheries | Fisheries Act 1982 Section 41 | DF 16/31 |
| Aboriginal Lands Council | Aboriginal Land Act 1980 | DHA/644-651 GAL/ 134-141 |
| RAN | Nil | Letter of permission |
| Conservation and Land Management (CALM) Permit for research/educational excursion | Conservation and Land Management Act 1984 Regulation 46 | NE000019 |
| Conservation and Land Management (CALM) Permit for research/educational excursion | Conservation and Land Management Act 1984 Reg 46 | NE000081 |
| Conservation and Land Management (CALM) Licence to take fauna for scientific purposes | Wildlife Conservation Act 1950 Regulation 17 | SF000144 |
| Rottnest Island Authority | Nil | Letter of permission |
| Fisheries Dept | Fisheries Act 1905 Section 20 | FD 526/75 V3 |
| Fisheries Dept | Fisheries Act 1905 Section 20 | FD 526/75 V4 |
| Fisheries Dept | Fisheries Act 1905 Section 20 | FD 526/75 V5 |
| GBRMPA | GBRMPA Regulation |  |
| GBRMPA | GBRMPA Regulation | G94/587 |
| GBRMPA | GBRMPA Regulation | G00/506 |
| NSW Fisheries | Fisheries Management Act 1994 | P03/0038 |
| GBRMPA | GBRMPA Regulation | G88/354 |
| Department of Primary Industries and Fisheries (Tasmania) | Fisheries Act 1959 | 95/96 - 27 |
| Australian Fisheries Management Authority | Fisheries Management Act 1991 | 900156 |
