# Supplementary material for: Phylogeny Drives Large Scale Patterns in Australian Marine Bioactivity and Provides a New Chemical Ecology Rationale for Future Biodiscovery
Source: PLoS One. 2013 Sep 5;8(9):e73800. doi: 10.1371/journal.pone.0073800 (PMC3763996; doi:10.1371/journal.pone.0073800)
Supplement: File S2 — Experimental detail for sample processing and biossays; and number of samples in each bioassay × bioregion × phylum combination. (DOCX) [file pone.0073800.s002.docx]

**Phylogeny drives large scale patterns in Australian marine bioactivity and provides a new chemical ecology rationale for future biodiscovery.**

**File S2: Experimental detail for sample processing and bioassays; and number of samples in each bioassay x bioregion x phylum combination.sample sizes.**

Approximately 2g of tissue (wet weight) from each sample was placed in a vial and freeze dried, then steeped in ethanol (10ml) for a minimum of 2 days. A 0.5 ml aliquot was removed and evaporated to dryness prior to 0.5 ml DMSO being added to reconstitute the sample for screening, concentration unknown. Extracts tested in the neuronal nitric oxide synthase (nNOS) assay were first pre-treated to remove arginine, which is the enzyme substrate and so had the potential to interfere with the assay. This was performed by diluting 1 ml of extract to 10% with water and applying to a 500 mg reverse-phase C_18_ sep-pak column (Alltech Associates) that was pre-conditioned with 5 volumes of methanol, water and 10% methanol/water respectively. The column was washed with 2ml 10% methanol and the extract to be tested eluted with 2ml 10% DMSO in methanol. All extracts were maintained at -20 ˚C until screened.

Several bioassays were included in this study (see experimental detail below), and were grouped into the following three functional categories for the purpose of further analysis of bioactivity: Cytotoxicity (against 3 human tumor cell lines); Anti-microbial activity (against 4 surrogates of microbial pathogens of humans); and CNS protection (inhibition of nNOS and the calcium channel). Tables 1-3 list the number of samples screened in all bioassays of each of these three categories within each phylum and bioregion.

*Cytotoxicity assays*

Cytotoxicity bioassay data analysed here was from one-dose bioassays conducted by the National Cancer Institute (<http://dtp.nci.nih.gov/>) according to the methodology of the Developmental Therapeutics Program, NCI/NIH. (<http://www.dtp.nci.nih.gov/branches/btb/ivclsp.html>). In summary, these bioassays measure cell growth, relative to a no-drug control and the time zero number of cells. All samples represented in table 1 were tested in three cell lines sourced from the National Cancer Intitute (NCI, Bethesda, MD): H460 (NCI-H460, carcinoma, non-small cell lung cancer); CEM (CCRF-CEM, acute lymphoblastic leukemia); and SF268 (SF-268, glioblastoma, central nervous system cancer)[[54](#_ENREF_54)].

*Anti-microbial assays*

All samples outlined in Table 2 were tested against four microbial strains: *Staphylococcus aureus* (ATCC 25923), *Escherichia coli* (ATCC 25922), *Mycobacterium smegmatis* (ATCC 14468) and *Candida albicans* (NCYC 1472. For each assay, a single colony was inoculated into 20 ml of sterile growth media (*S. aureus* - PYE media (peptone 10g/l, yeast extract 5g/l, NaCl 5g/l), *E. coli* - LB media (BD, Brisbane, Australia), *M. smegmatis* - Middlebrook 7H9 plus ADC enrichment (BD, Brisbane, Australia) and *C. albicans* - YM media (BD, Brisbane, Australia) and incubated overnight at 37°C. The initial inoculums was then diluted 1000 fold for extract testing in 96-well plates along with appropriate solvent and antibiotic controls (500 µg/ml ampicillin for *E. coli* and *S. aureus*, 500 µg/ml streptomycin for *M. smegmatis* and 6.25 µg/ml amphotericin B for *C. albicans*). Assays were incubated at 37°C overnight for *E. coli*, *S. aureus* and *C. albicans* or 3 days for *M. smegmatis*. Optical density readings at 595 nm were used to determine growth inhibition.

*CNS Protective Assays:*

Bioassays to measure inhibition of neuronal nitric oxide synthase (nNOS); and the N-type calcium channel, were conducted with all samples represented in Table 3. Inhibition of neuronal nitric oxide synthase (nNOS) was measured by monitoring the conversion of [^3^H]arginine to [^3^H]citrulline, according to the protocol described in [[55](#_ENREF_55)]. Blockage of the N-type Calcium channel was measured in a bioassay derived from that of [[56](#_ENREF_56)], except miniaturised to a 96-well plate plate format.

**Biota included in this study:**

Tables A-C provide the sample size of each bioregion/taxa combination screened in all bioassays in each bioassay category.

**Table A:** Numbers of unique samples per phyla and bioregion, included in each of the three cytotoxicity bioassays.

| **Phylum** | **Bioregion Number** | | | | | | | | | | **Total** |
| --- | --- | --- | --- | --- | --- | --- | --- | --- | --- | --- | --- |
|  | **1** | **2** | **3** | **4** | **5** | **6** | **7** | **8** | **10** | **11** |  |
| ANGIOSPERMATA |  | 4 | 5 | 5 | 28 | 0 | 3 | 2 | 1 | 8 | **56** |
| ANNELIDA | 2 | 1 | 6 | 2 | 0 | 0 | 0 | 1 | 4 | 1 | **17** |
| BRYOZOA | 12 | 8 | 6 | 9 | 5 | 4 | 6 | 3 | 5 | 7 | **65** |
| CHLOROPHYTA | 3 | 6 | 9 | 11 | 37 | 2 | 1 | 5 | 6 | 16 | **96** |
| CHORDATA | 24 | 23 | 31 | 26 | 65 | 25 | 34 | 31 | 27 | 47 | **333** |
| CNIDARIA | 24 | 14 | 12 | 53 | 244 | 43 | 29 | 19 | 23 | 8 | **469** |
| CRUSTACEA | 2 | 3 | 6 | 2 | 15 | 1 | 0 | 3 | 0 | 5 | **37** |
| CYANOPHYTA | 0 | 4 | 0 | 1 | 6 | 0 | 1 | 2 | 1 | 0 | **15** |
| ECHINODERMATA | 14 | 9 | 13 | 12 | 51 | 8 | 2 | 21 | 11 | 12 | **153** |
| MOLLUSCA | 11 | 8 | 31 | 10 | 57 | 7 | 5 | 11 | 6 | 24 | **170** |
| PHAEOPHYTA | 11 | 17 | 17 | 14 | 17 | 6 | 3 | 4 | 9 | 20 | **118** |
| PORIFERA | 263 | 120 | 66 | 75 | 215 | 130 | 125 | 87 | 118 | 110 | **1309** |
| RHODOPHYTA | 35 | 42 | 12 | 17 | 25 | 9 | 4 | 12 | 23 | 42 | **221** |
| **Grand Total** | **401** | **259** | **214** | **237** | **765** | **235** | **213** | **201** | **234** | **300** | **3059** |

**Table B:** Numbers of unique samples per phyla and bioregion, included in each of the four anti-microbial bioassays.

| **Phylum** | **Bioregion Number** | | | | | | | | | | **Total** |
| --- | --- | --- | --- | --- | --- | --- | --- | --- | --- | --- | --- |
|  | **1** | **2** | **3** | **4** | **5** | **6** | **7** | **8** | **10** | **11** |  |
| ANGIOSPERMATA | 0 | 5 | 6 | 6 | 54 | 0 | 13 | 3 | 2 | 8 | **97** |
| ANNELIDA | 3 | 1 | 12 | 30 | 42 | 0 | 18 | 12 | 4 | 1 | **123** |
| BRYOZOA | 20 | 13 | 16 | 51 | 85 | 5 | 48 | 37 | 8 | 8 | **291** |
| CHLOROPHYTA | 7 | 12 | 9 | 28 | 133 | 1 | 20 | 16 | 9 | 27 | **262** |
| CHORDATA | 33 | 28 | 56 | 157 | 571 | 20 | 159 | 148 | 44 | 66 | **1282** |
| CNIDARIA | 15 | 15 | 24 | 167 | 766 | 19 | 278 | 157 | 34 | 12 | **1487** |
| CRUSTACEA | 10 | 3 | 10 | 61 | 101 | 0 | 146 | 38 | 1 | 6 | **376** |
| CYANOPHYTA | 0 | 4 | 0 | 2 | 13 | 0 | 1 | 4 | 1 | 0 | **25** |
| ECHINODERMATA | 26 | 12 | 29 | 128 | 320 | 2 | 252 | 127 | 19 | 17 | **932** |
| MOLLUSCA | 15 | 11 | 49 | 120 | 240 | 5 | 109 | 52 | 14 | 36 | **651** |
| PHAEOPHYTA | 17 | 33 | 22 | 23 | 57 | 5 | 14 | 15 | 15 | 32 | **233** |
| PORIFERA | 209 | 130 | 154 | 272 | 1479 | 103 | 444 | 412 | 175 | 167 | **3545** |
| RHODOPHYTA | 33 | 71 | 17 | 34 | 103 | 7 | 24 | 38 | 31 | 62 | **420** |
| **Grand Total** | **388** | **338** | **404** | **1079** | **3964** | **167** | **1526** | **1059** | **357** | **442** | **9724** |

**Table C:** Numbers of unique samples per phyla and bioregion, included in both of the two CNS-protective bioassays.

| **Phylum** | **Bioregion Number** | | | | | | | | | | Total |
| --- | --- | --- | --- | --- | --- | --- | --- | --- | --- | --- | --- |
|  | **1** | **2** | **3** | **4** | **5** | **6** | **7** | **8** | **10** | **11** |  |
| ANGIOSPERMATA | 0 | 1 | 0 | 5 | 12 | 0 | 5 | 0 | 1 | 1 | 25 |
| ANNELIDA | 3 | 1 | 6 | 28 | 43 | 0 | 15 | 9 | 0 | 0 | 105 |
| BRYOZOA | 10 | 10 | 10 | 41 | 54 | 3 | 33 | 23 | 0 | 1 | 185 |
| CHLOROPHYTA | 5 | 4 | 3 | 23 | 69 | 0 | 17 | 4 | 2 | 1 | 128 |
| CHORDATA | 27 | 23 | 26 | 120 | 277 | 4 | 53 | 60 | 11 | 12 | 613 |
| CNIDARIA | 10 | 8 | 12 | 120 | 594 | 5 | 153 | 69 | 4 | 0 | 975 |
| CRUSTACEA | 9 | 1 | 5 | 58 | 81 | 0 | 101 | 35 | 0 | 0 | 290 |
| CYANOPHYTA | 0 | 2 | 0 | 1 | 6 | 0 | 0 | 1 | 0 | 0 | 10 |
| ECHINODERMATA | 20 | 5 | 15 | 123 | 294 | 2 | 185 | 76 | 3 | 1 | 724 |
| MOLLUSCA | 10 | 5 | 21 | 98 | 197 | 1 | 84 | 29 | 4 | 10 | 459 |
| PHAEOPHYTA | 9 | 11 | 8 | 19 | 29 | 1 | 9 | 6 | 5 | 5 | 102 |
| PORIFERA | 118 | 70 | 63 | 203 | 836 | 39 | 198 | 162 | 38 | 34 | 1761 |
| RHODOPHYTA | 16 | 20 | 4 | 20 | 53 | 3 | 13 | 8 | 6 | 19 | 162 |
| **Grand Total** | **237** | **161** | **173** | **859** | **2545** | **58** | **866** | **482** | **74** | **84** | 5539 |
